# Rapid detection of pathological mutations and deletions of the haemoglobin beta gene (*HBB*) by High Resolution Melting (HRM) analysis and Gene Ratio Analysis Copy Enumeration PCR (GRACE-PCR)

**DOI:** 10.1186/s12881-016-0334-y

**Published:** 2016-10-19

**Authors:** Andrew Turner, Jurgen Sasse, Aniko Varadi

**Affiliations:** 1 Department of Pathology and Laboratory Medicine, Sheikh Khalifa Medical City, Abu Dhabi, United Arab Emirates; 2 Department of Applied Sciences, Faculty of Health and Applied Sciences, University of the West of England, Bristol, UK

**Keywords:** Beta thalassaemia, Copy number determination, Gene quantification, HRM, GRACE-PCR

## Abstract

**Objectives:**

Inherited disorders of haemoglobin are the world’s most common genetic diseases, resulting in significant morbidity and mortality. The large number of mutations associated with the haemoglobin beta gene (*HBB*) makes gene scanning by High Resolution Melting (HRM) PCR an attractive diagnostic approach. However, existing HRM-PCR assays are not able to detect all common point mutations and have only a very limited ability to detect larger gene rearrangements. The aim of the current study was to develop a *HBB* assay, which can be used as a screening test in highly heterogeneous populations, for detection of both point mutations and larger gene rearrangements.

**Methods:**

The assay is based on a combination of conventional HRM-PCR and a novel Gene Ratio Analysis Copy Enumeration (GRACE) PCR method. HRM-PCR was extensively optimised, which included the use of an unlabelled probe and incorporation of universal bases into primers to prevent interference from common non-pathological polymorphisms. GRACE-PCR was employed to determine *HBB* gene copy numbers relative to a reference gene using melt curve analysis to detect rearrangements in the *HBB* gene. The performance of the assay was evaluated by analysing 410 samples.

**Results:**

A total of 44 distinct pathological genotypes were detected. In comparison with reference methods, the assay has a sensitivity of 100 % and a specificity of 98 %.

**Conclusion:**

We have developed an assay that detects both point mutations and larger rearrangements of the *HBB* gene. This assay is quick, sensitive, specific and cost effective making it suitable as an initial screening test that can be used for highly heterogeneous cohorts.

## Background

According to the World Health Organization (WHO), inherited disorders of haemoglobin are the most common monogenic disorders in the world. The WHO estimates that 300,000 to 400,000 severely affected infants are born each year and that greater than 95 % of these severe cases arise from genetic abnormalities of the β-globin gene (*HBB*) [1]. Globally, these conditions result in significant childhood morbidity and mortality.

The *HBB* gene is 1606 base pairs long, contains three exons and is located on the short arm of chromosome 11 towards the 3’ end of a 70 kb region known as the β-globin locus. The *HBB* gene codes for β-globin, a protein that is an essential component of normal adult haemoglobin [2]. To date, 868 mutations of the *HBB* gene have been described that have either a qualitative or quantitative effect on β-globin synthesis. These include point mutations, small insertions / deletions (indels) and larger gene rearrangements [3]. Qualitative defects are normally the result of point mutations and lead to the formation of abnormal β-globin chains, which in turn give rise to variant haemoglobins. Quantitative defects of the *HBB* gene either reduce, or totally stop, the synthesis of β-globin resulting in β-thalassaemia. Unlike α-thalassaemia, which is mostly caused by large deletions, the majority of β-thalassaemia alleles are the result of point mutations or small indels. However, in recent years several novel deletions of the *HBB* gene have been identified and it has become apparent that deletional forms of β-thalassaemia are more prevalent than was previously thought. Indeed, it has been suggested that large deletions may account for as much as 10 % of β-thalassaemia mutations [4].

Premarital screening programs specifically targeting β-thalassaemia were introduced in Greece [5] and Cyprus [6, 7] in the 1970s. These programs significantly reduced the number of children being born with β-thalassaemia, and as a result similar programs were subsequently introduced in a number of countries where the disease is prevalent [8]. The success of such premarital screening programs is dependent on reliable laboratory tests for the detection of carriers.

In general, carriers for the common variant haemoglobins are readily detected by routine biochemical techniques, such as cation exchange High Performance Liquid Chromatography (HPLC) or capillary electrophoresis (CE). However, reliable detection of β-thalassaemia carriers can be more challenging [9]. Haemoglobin A_2_ (Hb A_2_) quantification is widely used for screening, since Hb A_2_ is easily quantified by HPLC or CE and shows a modest increase in the majority of individuals with β-thalassaemia trait [10]. However, some β-thalassaemia mutations result in only minimal increases in Hb A_2_ levels and are easily missed during screening [11]. Interpretation of β-thalassaemia screening results can be further complicated by external factors that may influence the Hb A_2_ level. For example, Hb A_2_ is lowered by both iron deficiency [12] and α-thalassaemia [13], but is elevated by mutations in the Krüppel-like Factor 1 (*KLF1*) gene [14]. In addition, co-inheritance a of β-thalassaemia trait with a δ-globin gene (*HBD*) mutation can lead to Hb A_2_ levels that are normal or even reduced [11]. Consequently, although Hb A_2_ quantification remains a useful screening tool, confirmation by molecular analysis is frequently required [15].

A variety of PCR techniques, such as Amplification Refractory Mutation System (ARMS) PCR, Allele Specific Oligonucleotide (ASO) PCR and real time PCR have been used to detect β-thalassaemia resulting from point mutations or indels [16–18], while Gap-PCR is normally used to detect larger gene rearrangements [4]. These assays are designed to detect either a single specific mutation or a small set of mutations. This approach works well in populations where a limited number of β-thalassaemia mutations are prevalent, but is less useful when testing highly heterogeneous populations where the range of mutations is less predictable. In such situations gene scanning methods may be useful and assays using various technologies including Denaturing HPLC (DHPLC), Single Strand Conformational Polymorphism (SSCP) and Denaturing Gradient Gel Electrophoresis (DGGE) have been described [19]. These assays offer an alternative means of detecting point mutations or indels of the *HBB* gene, but are not appropriate for the detection of larger deletions and have the disadvantage of being open tube techniques that require significant post amplification processing.

An alternative gene scanning technology called High Resolution Melting PCR (HRM-PCR) offers potential advantages over the other techniques since it does not require complicated post amplification processing by chromatography or polyacrylamide gel electrophoresis [20]. HRM-PCR is a closed tube technique that allows both amplification and detection to be performed on the same instrument without requiring further hands-on time after the initial PCR setup [20]. Thus, HRM-PCR is well suited to clinical applications, since it permits a simple and efficient workflow, with minimal risk of amplicon contamination. In addition to being quicker and easier to perform, HRM-PCR has specificity and sensitivity equal to, or better than, other gene scanning technologies such as DHPLC [21, 22]. These advantages have resulted in the application of HRM-PCR in a wide range of clinical settings [23–28].

HRM-PCR assays for scanning the *HBB* gene mutations have been described, targeting the most common mutations found in Chinese [29] and South East Asian [30, 31] populations. These assays are well suited for screening the specific cohorts targeted. However, the approaches used to overcome interference from high frequency single nucleotide polymorphisms (SNPs) made these assays insensitive to some important pathological mutations that are common in other populations including Hb S and Hb C. Additionally, with the exception of the 3.4 kb deletion, these assays cannot detect larger gene rearrangements [31].

We have recently developed a Gene Ratio Copy Enumeration PCR (GRACE-PCR) to determine copy numbers for the α-globin genes [32] and we describe a similar approach for the detection of deletions of the *HBB* gene. We also developed a HRM-PCR method that was optimized with the use of an unlabelled probe and primers containing universal bases to address the issue of SNP interference. The combined use of GRACE-PCR and HRM-PCR allowed for the detection of point mutations, indels and larger deletions of the *HBB* gene, thus resulting in an assay that is universally applicable.

## Materials and methods

### Samples and cohort selection

This study was conducted using anonymous, archived material from blood specimens submitted to the Sheikh Khalifa Medical City (SKMC) laboratory, Abu Dhabi, United Arab Emirates (UAE) for thalassaemia/haemoglobinopathy screening. Ethical clearance was obtained from the SKMC Institutional Research and Ethics Committee to use this material for the current study. Since the study used anonymous archived material it was not possible to identify the subjects, therefore specific informed consent was not possible. The material used contained no patient identifiers, but the results of the haemoglobin analysis by HPLC had been assigned prior to the anonymization of samples. A total of 410 samples were used in the study, these included 305 with elevated Hb A_2_ (Hb A_2_ > 3.5 %), 27 with low or normal Hb A_2_, 76 with abnormal haemoglobins and two samples known to be positive for the 619 base pair deletion (NM_000518.4:c.316-149_*342delinsAAGTAGA) [33]. The initial assessment of the assay was conducted using 342 of these samples and an additional 68 were used for a blinded validation.

### DNA extraction

Genomic DNA was extracted from whole blood using the QIAamp DNA Blood Mini kit (Qiagen, Germany) in accordance with the manufacturer’s instructions. The concentration of the extracted DNA was measured using a NanoDrop 2000 spectrophotometer (Thermo Scientific, USA) and adjusted to 10 ng/μl with 10 mM Tris, 0.5 mM EDTA (pH 9.0).

### Primer design and synthesis

**Fig. 1:**
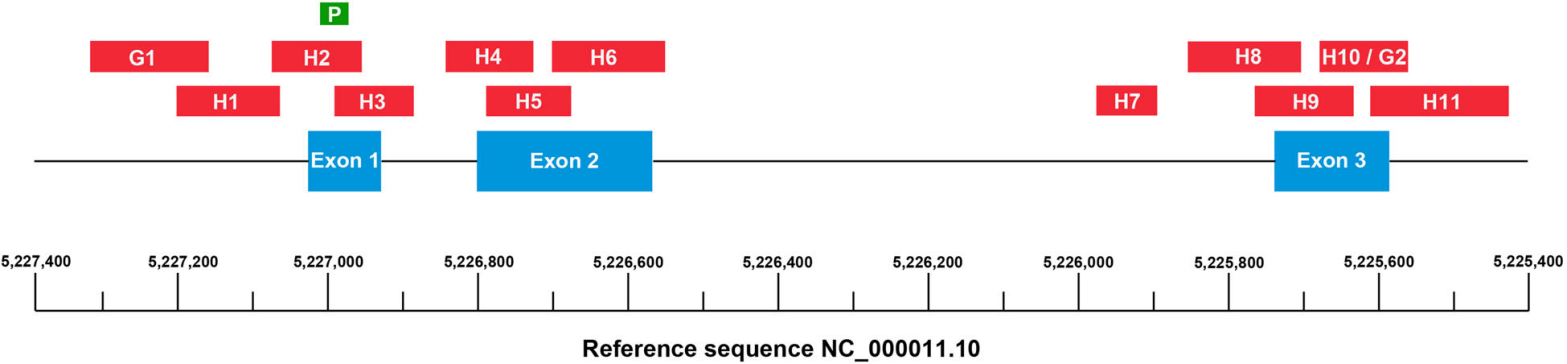
Schematic representation the *HBB* gene showing amplicons and probe positions. A total of 11 primer pairs were used to scan the *HBB* gene by HRM-PCR (H1 to H11). In addition two primer pairs where used to test for deletions by GRACE-PCR (G1 and G2). The unlabelled probe (indicated by P) was used in conjunction with primer set H2. Primer sets H6 and H7 incorporated the universal base inosine in place of common non-pathological SNPs (See sequence information in Table 1)

Analysis of the HbVar online database (http://globin.bx.psu.edu/hbvar/menu.html) was performed to identify the regions of the *HBB* gene of potential clinical relevance and to identify the breakpoints for known gene deletions. The final version of the assay used a total of 13 PCR reactions, 11 HRM-PCRs to scan for point mutations and indels of the *HBB* gene and two GRACE-PCRs for the detection of larger gene rearrangements (Table 1 and Fig. 1).

**Table 1:** Primers for the HRM-PCR/GRACE-PCR assay of the *HBB* gene

Primer set	Direction	Primer/Probe sequence	Position on ref sequence	Product length (bp)	Target	Annealing temp (°C)	HRM melt range (°C)
H1	Forward	5’-GGCTGTCATCACTTAGACCTCA-3’	5,227,065 to 5,227,196 on NC_000011.10	132	*HBB* Promoter	59	80–90
	Reverse	5’-CAAATGTAAGCAATAGATGGCTC-3’					
H2	Forward	5’-ATTTGCTTCTGACACAACTG-3’	5,226,952 to 5,227,069 on NC_000011.10	118	*HBB* Exon 1	55	70–90
	Reverse	5’-CTTCATCCACGTTCACCTTG-3’					
	Probe^a^	5’-CTCCTGAGGAGAAGTCT-GCCGTTACTGCCCTGTGGGG-3’					
H3	Forward	5’-TGCCGTTACTGCCCTGT-3’	5,226,886 to 5,226,992 on NC_000011.10	107	*HBB* Exon 1	59	75–85
	Reverse	5’-TTCTATTGGTCTCCTTAAACCTGT-3’					
H4	Forward	5’-CACTGACTCTCTCTGCCTA-3’	5,226,726 to 5,226,841 on NC_000011.10	116	*HBB* Exon 2	59	80–90
	Reverse	5’-TAACAGCATCAGGAGTGGACA-3’					
H5	Forward	5’-GTCTACCCTTGGACCCAG-3’	5,226,674 to 5,226,789 on NC_000011.10	116	*HBB* Exon 2	59	80–90
	Reverse	5’-CTAAAGGCACCGAGCACT-3’					
H6	Forward	5’-GCTCATGGCAAGAAAGTGCTC-3’	5,226,549 to 5,226,705 on NC_000011.10	157	*HBB* Exon 2	59	80-90
	Reverse^b^	5’-GAAAACATCAAGIGTCCCA-3’					
H7	Forward	5’-TGCCTCTTTGCACCATTCTA-3’	5,225,893 to 5,225,974 0n NC_000011.10	82	*HBB* IVS2	55	70–80
	Reverse^b^	5’-GAAATATTTATATGCAGAIATATTGCTA-3’					
H8	Forward	5’-CTAATAGCAGCTACAATCCAG-3’	5,225,702 to 5,225,852 on NC_000011.10	152	*HBB* IVS2	56	80–90
	Reverse	5’-CACAGACCAGCACGTT-3’					
H9	Forward	5’-TTGCTAATCATGTTCATACCTC-3’	5,225,632 to 5,225,765 on NC_000011.10	134	*HBB* Exon 3	59	80–90
	Reverse	5’-CCAGCCACCACTTTCTGAT-3’					
H10/G2	Forward	5’-GAATTCACCCCACCAGTGC-3’	5,225,559 to 5,225,678 on NC_000011.10	120	*HBB* Exon 3	55	80–90
	Reverse	5’-AGGAACCTTTAATAGAAATTGGAC-3’					
H11	Forward	5’-CCCACAAGTATCACTAAGCTC-3’	5,225,423 to 5,225,614 on NC_000011.10	192	*HBB* Exon 3	55	78–88
	Reverse	5’-CCCTTTTTAGTAAAATATTCAGA-3’					
G1	Forward	5’-TGAAGTCCAACTCCTAAGCC-3’	5,227,086 to 5,227,243 on NC_000011.10	158	*HBB* Promoter	55	75–90
	Reverse	5’-TCTGCCCTGACTTTTATGCC-3’					
G3	Forward	5’-CACCCGGCCTCATGGAT-3’	1,462,101 to 1,462,255 on NC_000016.10	155	*CLCN7* control for GRACE PCR	55	NA
	Reverse	5’-AAGAGAACTACAGACCAACACCC-3’					

Primer sets H1 to H11 were used to scan the *HBB* gene for mutations by HRM. Primers sets G1 and G2 were used to detect deletions of the *HBB* gene promoter and third exon by GRACE-PCR. Primer set G3 is the internal control for the GRACE-PCR reactions

^a^The probe used in conjunction with primer set H2 is an unlabelled oligonucleotide that has a phosphate block at the 3’ end

^b^The reverse primers of primer sets H6 and H7 contain the universal base inosine, indicated with an I in the primer sequence

The previously described primer set H7 [29] was included to test for the HBB:c.316-197C > T mutation found in intervening sequence two (IVS2). The reverse primers in primer sets H6 and H7 incorporated the universal base inosine to eliminate the risk of potential interference from common non-pathological SNPs (Table 1). In addition, an unlabelled probe was designed to overlap the region of the first exon containing the HBB:c.19G > A and HBB:c.20A > T mutations (Fig. 1 and Table 1). This probe was designed to have a melting temperature approximately 8 °C lower than the corresponding amplicon and was synthesized with a phosphate block at the 3’ prime end to prevent it from acting as a primer.

Primer sets G1 and G2 were used to amplify targets in the *HBB* gene promoter and third exon, respectively. These primers were designed to be used in GRACE-PCR with primer set G3, which amplifies a target in the *CLCN7* reference gene [34].

All primers were checked for specificity using the NCBI Primer BLAST program (http://www.ncbi.nlm.nih.gov/tools/primer-blast). HPLC purified primers and probes were commercially synthesized (Metabion, Germany).

### PCR reaction conditions

For scanning the *HBB* gene by HRM with primers sets H1 to H11 each 10 μl HRM-PCR reaction included 5 μL of LightCycler® 480 High Resolution Melting Master (Roche Diagnostics, Germany), 3.75 mM MgCl_2_, 25 ng of genomic DNA and 0.2 μM of each primer. When the unlabelled probe was included, it was used at a concentration of 0.2 μM and the forward primer concentration was reduced to 0.02 μM. PCR was performed using a Rotor-Gene Q-5plex HRM thermocycler (Qiagen). This system can accommodate up to 72 PCR reactions per run. After a 10 min hold at 95 °C, 45 cycles of PCR were performed as follows: 15 s at 95 °C, 15 s at the temperature given in Table 1, 15 s at 72 °C. Cycling was followed by a 2 min hold at 72 °C, a 1 min hold at 95 °C and then a 1 min hold at 40 °C. Amplicon melting was performed at a rate of 0.1 °C/s over the temperature ranges indicated in Table 1.

For the detection of deletions in the promoter of the *HBB* gene promoter, each 12.5 μL GRACE-PCR reaction included 0.25 units of Kappa HiFi HotStart polymerase (Kappa Biosystems, USA), 0.25 units of Platinum Taq polymerase (Invitrogen, USA), 1x Kappa GC buffer (Kappa Biosystems), 0.3 mM of each dNTP (Kappa Biosystems), 0.25 μL Resolite dye (Roche Diagnostics), 25 ng of genomic DNA, 0.16 μM of each G1 primer and 0.1 μM of each G3 primer (Table 1). After a 2 min hold at 98 °C, 26 cycles of PCR were performed as follows: 15 s at 95 °C, 15 s at 55 °C, 15 s at 72 °C. Melting was performed from 75 to 90 °C at a rate of 0.2 °C/s.

For the detection of deletions in the third exon of the *HBB* gene, each 10 μL GRACE-PCR reaction included 5 μL of LightCycler® 480 High Resolution Melting Master (Roche Diagnostics), 3.0 mM MgCl_2_, 25 ng of genomic DNA, 0.2 μM of each G2 primer and 0.08 μM of each G3 primer (Table 1). After a 10 min hold at 95 °C, 26 cycles of PCR were performed as follows: 15 s at 95 °C, 15 s at 55 °C, 15 s at 72 °C. Melting was performed from 80 to 90 °C at a rate of 0.2 °C/s.

### Acquisition and analysis of data

**Fig. 2:**
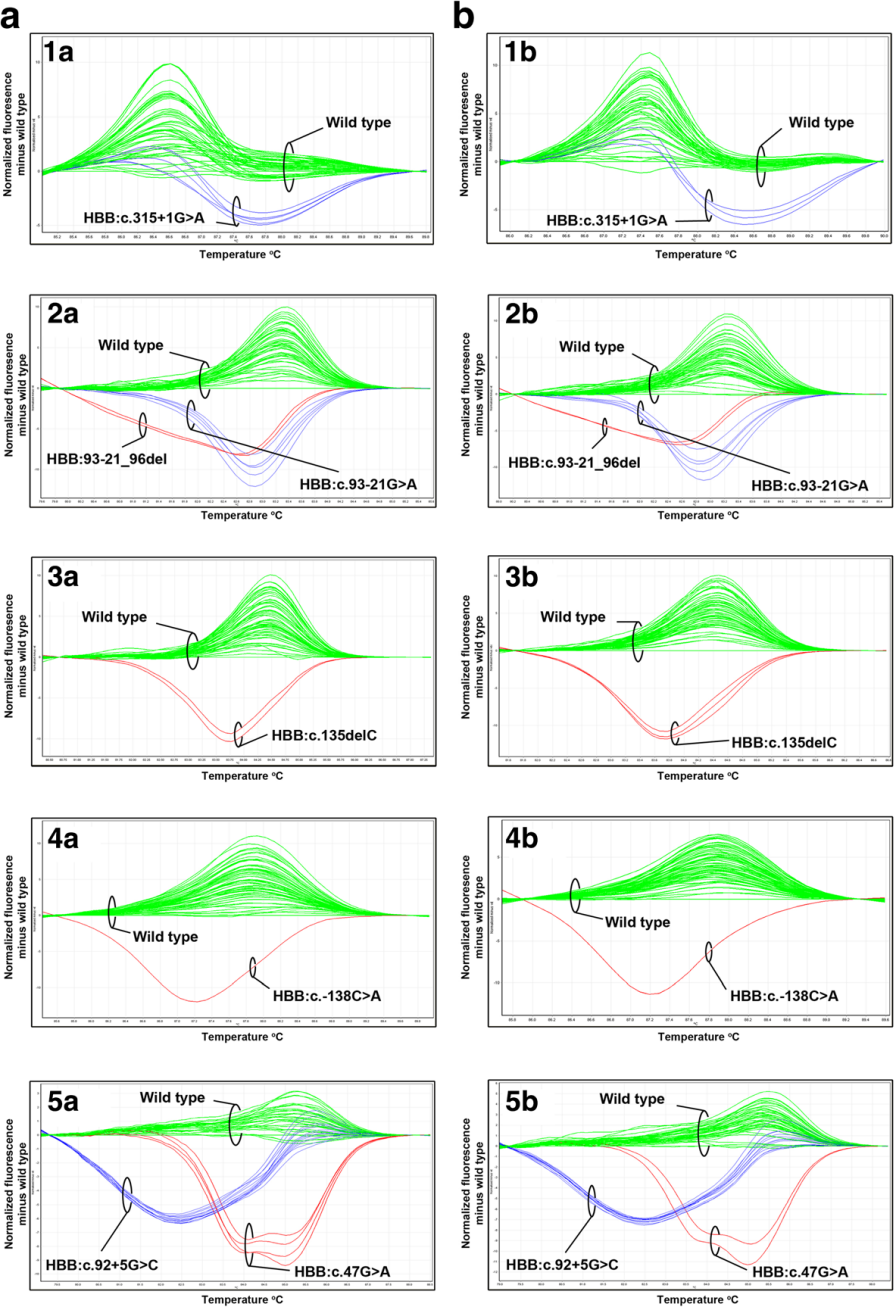
Representative HRM-PCR difference plots. Difference plots show wild type samples and the various mutants: 1a&b – genotype 37; 2a&b – genotype 28; 3a&b – genotype 33; 4a&b – genotype 2; 5a&b – genotypes 24 and 18 (see Table 3). Plots 1a&b were generated using primer pair 6 containing the universal base inosine (Table 1). Panels **a** and **b** show different runs with different samples

**Fig. 3:**
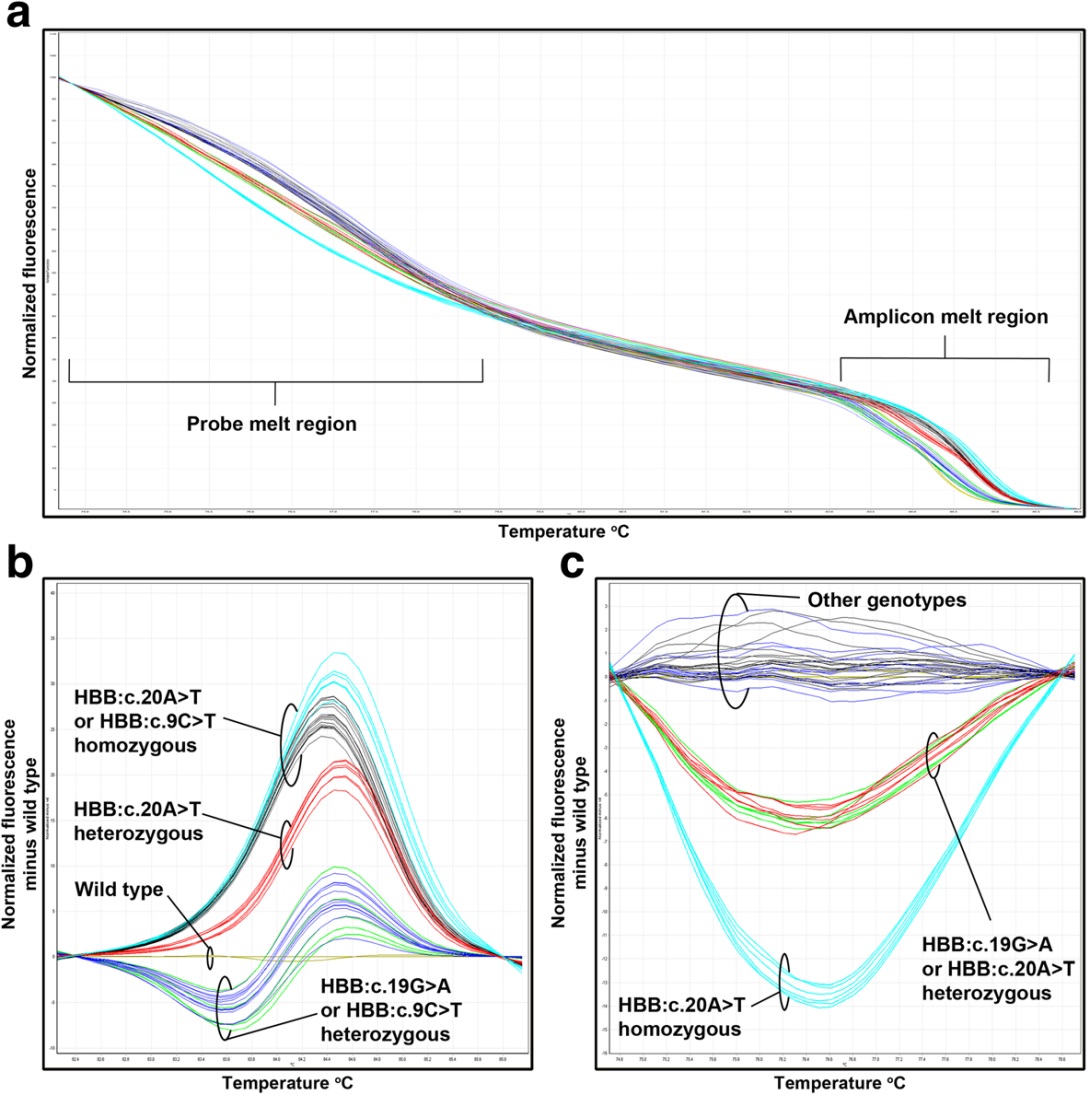
Representative unlabelled probe HRM-PCR difference plots. Addition of the unlabeled probe allows differentiation of the pathological HBB:c.19G > A heterozygotes from common non-pathological HBB:c.9C > T heterozygotes; and of HBB:c.20A > T homozygotes from HBB:c.9C > T homozygotes. The unlabeled probe produced distinct probe and amplicon melt regions (**a**). Certain pathological variants cannot be distinguished from the HBB:c.9C > T SNP in the amplicon melt region (**b**), but are clearly separated in the probe melt region (**c**)

**Fig. 4:**
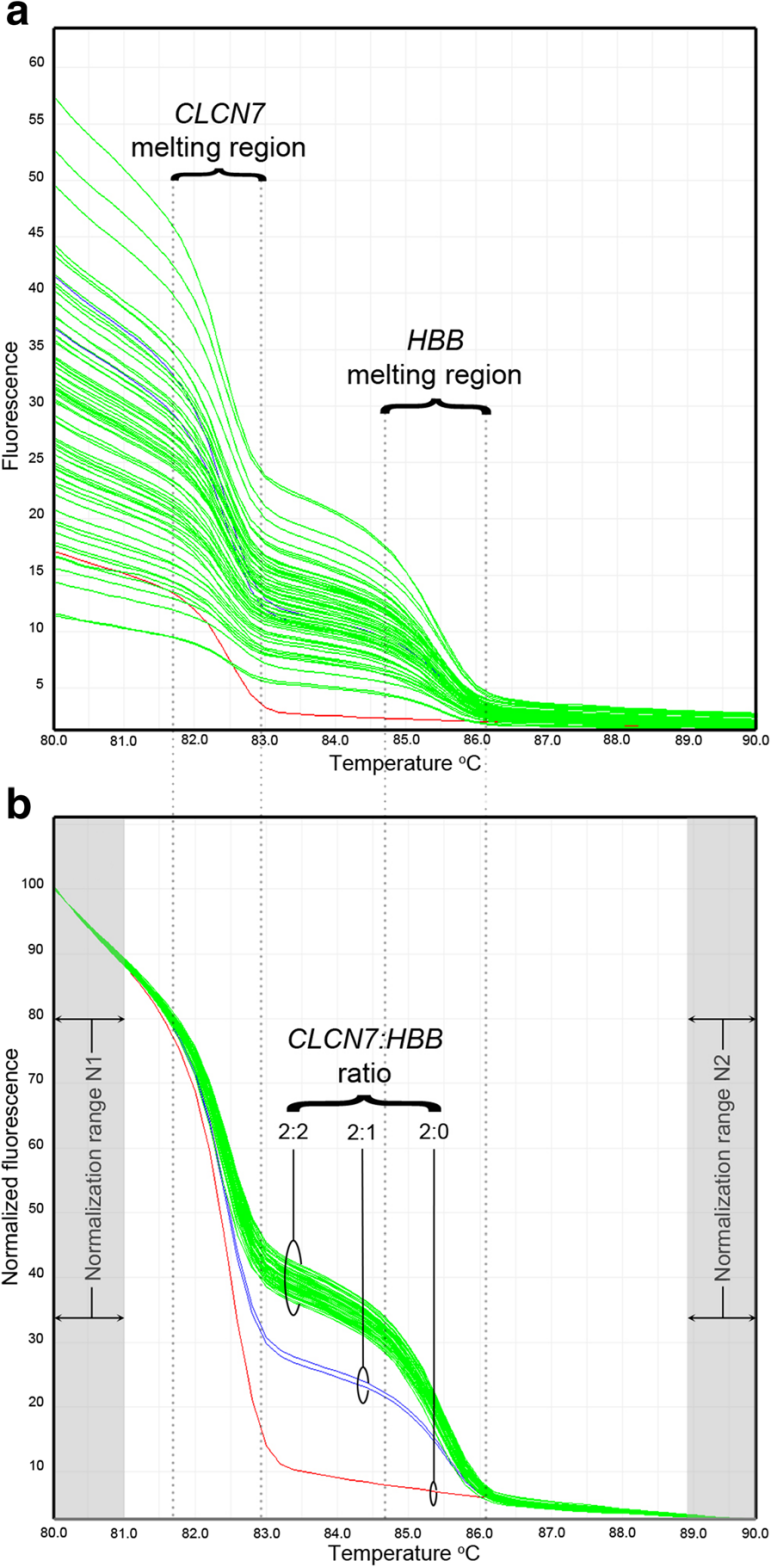
GRACE-PCR to determine copy numbers of the *HBB* gene. Limited cycle GRACE-PCR was used to amplify targets from the *CLCN7* reference gene [34] and the *HBB* gene. The raw melt curves (**a**) are difficult to interpret, but after normalization (**b**) the ratio of *CLCN7:HBB* gene copy numbers is easily visualized

Data acquisition and analysis were performed using the Rotor-Gene Q series software version 1.7 (Qiagen). For gene scanning by HRM, the raw melting curves were normalized and then converted to difference plots (Figs. 2 and 3). For the determination of gene copy number by GRACE-PCR, the melt curves were normalized before the melting of the *CLCN7* control gene and after the *HBB* gene (Fig. 4). This resulted in a plot which allowed the *CLCN7:HBB* ratio to be determined by simple visual examination (Fig. 4b). Since the copy number of the *CLCN7* gene is assumed to be two, the copy number of the *HBB* gene is easily derived.

### Confirmation of deletions in the *HBB* gene

The Filipino β-thalassaemia deletion (NG_000007.3:g.66258_184734del118477) [3, 35] was confirmed by Gap-PCR using previously described primers [36]. Each 12.5 μL PCR reaction included 0.25 units of Platinum Taq, 1x PCR buffer (Invitrogen), 0.3 mM of each dNTP, 2.0 mM MgCl_2_, 0.1 μM of each primer and 25 ng of genomic DNA (Table 2). After an initial 4 min hold at 94 °C, 38 cycles of PCR was performed as follows: 30 s at 94 °C, 30 s at 60 °C, 60 s at 72 °C. PCR products were evaluated by electrophoresis using 1 % agarose gels, with detection of a 920 bp product indicating the presence of the deletion.

**Table 2:** Primers used to generate amplicons for sequencing and Gap-PCR

Target	Direction	Primer sequence	Position on reference sequence	Product length (bp)	Annealing temp °C	Extension time (sec)
*HBB* Exon 1 and 2	Forward	5’-TGTCATCACTTAGACCTCACCCTG-3’	5,226,544 to 5,227,193 on NC_0000.11.10	686	58	40
	Reverse	5’-GGAAAGAAAACATCAAGCGTCCCATAG-3’				
*HBB* Intervening sequence 2	Forward	5’-TGCACGTGGATCCTGAGAACTTCA-3’	5,226,411 to 5,226,602 on NC_000011.10	228	56	20
	Reverse	5’-ACAGCAAATAAAAGAAACTAAAACGA-3’				
*HBB* Exon 3	Forward	5’-GCTGGATTATTCTGAGTCCAAGCTA-3’	5,225,508 to 5,225,797 on NC_000011.10	326	58	25
	Reverse	5’-TCAAGGCCCTTCATAATATCCCC-3’				
GAP-PCR Filipino β-thalassaemia deletion	Forward	5’-GTAAATGAGTAAATGAAGGAATGAT-3’	5,112,653 to 5,232,062 on NC_000011.10	920	60	60
	Reverse	5’-TGTGATTTGGCTCTCTTCTTGTCTA-3’				

The primers used to generate amplicons for sequencing the *HBB* gene are shown along with the appropriate PCR reaction conditions. The sequencing primers were synthesized with M13 tails. Primers used for the detection of the Filipino β-thalassaemia deletion by Gap-PCR are also shown (without M13 tails)

The presence of the 619 bp deletion of the *HBB* gene was confirmed using the commercial β-Globin StripAssay (ViennaLab Diagnostics, Austria) in accordance with the manufacturer’s instructions.

### Sequencing of the *HBB* gene

Amplicons for sequencing were obtained using the primers and reaction conditions given in Table 2. The PCR products were diluted 1:150 with distilled water prior to cycle sequencing. Cycle sequencing was performed with the BigDye Terminator v3.1 Cycle Sequencing kit (Applied Biosystems, USA) in accordance with the manufacturer’s instructions using M13 primers. Cycle sequencing products were cleaned by an ethanol and ethylenediaminetetraacetic acid (EDTA) precipitation and sequenced on a 3130 Genetic Analyser (Applied Biosystems).

## Results

### Amplicon and primer design

When a common non-pathological SNP was present in a region that was optimal for primer placement, we substituted the nucleotide at the SNP position with the universal base inosine. Incorporating inosine into the primer allows equal amplification of both the wild-type and variant alleles [37]. This proved to be a good strategy for obtaining clear HRM-PCR profiles without interference by the non-pathological SNP. The SNPs concerned were rs10768683 (HBB:c.315 + 16G > C) under the reverse H6 primer and rs1609812 (HBB:c.316-185C > T) under the reverse H7 primer (Table 1). An example showing the detection of the HBB:c.315 + 1G > A mutant and wild type alleles where inosine in the primer was used to mask the non-pathological SNP rs10768683 is shown in Fig. 2, plots 1A and 1B, and Table 3 (genotype 37).

**Table 3:** Mutations of the *HBB* gene detected by the HRM-PCR assay and GRACE-PCR

Genotype	Common name	Sequencing or Gap-PCR	Sample set 1	Sample set 2	Primer set used for detection	Phenotype
		(*n* = 410)	(*n* = 342)	(*n* = 68)		
1. HBB:c.-151C > T	-101 C > T	2	1	1^a^	H1	β^+^ thalassaemia trait
2. HBB:c.-138C > A	-88 C > A	5	3	2^a^	H1	β^+^ thalassaemia trait
3. HBB:c.-138C > A; HBB:c.92 + 5G > C	-88 C > A; IVS1-5 G > C	1	1	0	H1 + H3	β thalassaemia major
4. HBB:c.-121C > T	-71C > T	1	1	0	H1	β^+^ thalassaemia trait
5. HBB:c.17_18delCT^b^	Codon 5-CT	13	12	1	H2	β^o^ thalassaemia trait
6. HBB:c.17_18delCT; HBB:c.92 + 5G > C^b^	Codon 5 -CT; IVS1-5 G > C	1	1	0	H2 + H3	β thalassaemia major
7. HBB:c.19G > A	Hb C	7	5	2	H2	Hb C trait
8. HBB:c.19G > A; HBB:c.20A > T	Hb C; Hb S	1	0	1^a^	H2	Hb SC disease
9. HBB:c.20A > T heterozygote^b^	Hb S	24	21	3	H2	Hb S trait
10. HBB:c.20A > T homozygote^b^	Hb S	18	18	0	H2	Hb S disease
11. HBB:c.20A > T;HBB:c.92 + 5G > C^b^	Hb S; IVS1-5 G > C	3	3	0	H2 + H3	Hb S disease
12. HBB:c.20A > T; HBB:c.83-22_95del^b^	Hb S; 25 bp deletion	2	2	0	H2 + H4	Hb S disease
13. HBB:c.20A > T; HBB:c.176C > A	Hb S; Hb Sheffield	1	1	0	H2 + H5	Hb S + Hb Sheffield
14. HBB:c.20A > T; HBB:c.364G > C	Hb S; Hb D	2	2	0	H2 + H7	Hb SD disease
15. HBB:c.25_26delAA^b^	Codon 8 -AA	3	3	0	H2	β^o^ thalassaemia trait
16. HBB:c.27_28insG^b^	Codon 8/9 + G	5	4	1^a^	H2	β^o^ thalassaemia trait
17. HBB:c.33C > A; HBB:c.51delC	Codon 10 C > A; Codon 16 -C	1	1	0	H2 + H3	β thalassaemia major
18. HBB:c.47G > A^b^	Codon 15 G > A	6	4	2^a^	H2	β^o^ thalassaemia trait
19. HBB:c.67G > T	Codon 22 G > T	2	2	0	H3	β^o^ thalassaemia trait
20. HBB:c.79G > A	Hb E	7	5	2	H3	Hb E trait
21. HBB:c.79G > A; HBB:c.126_129delCTTT	Hb E; Codon 41/42 -CTTT	2	1	1	H3 + H4 + H5	Hb E/β^o^ thalassaemia
22. HBB:c.92G > C; HBB:c.-92C > G	Hb Monroe	3	1	2^a^	H1 + H3	β^o^ thalassaemia trait
23. HBB:c.92 + 1G > A^b^	IVS1-1 G > A	16	12	4	H3	β^o^ thalassaemia trait
24. HBB:c.92 + 5G > C^b^	IVS1-5 G > C	96	82	14	H3	β^+^ thalassaemia trait
25. HBB:c.92 + 6 T > C^b^	IVS1-6 T > C	7	6	1^a^	H3	β^+^ thalassaemia trait
26. HBB:c.92 + 6 T > C; HBB:c.93-21_96del^b^	IVS1-6 T > C; 25 bp deletion	1	1	0	H3 + H4	β thalassaemia major
27. HBB:c.93-21_96del^b^	25 bp deletion	35	27	8	H4	β^o^ thalassaemia trait
28. HBB:c.93-21G > A^b^	IVS1-110 G > A	18	11	7	H4	β^+^ thalassaemia trait
29. HBB:c.112delT^b^	Codon 36/37 -T	1	1	0	H4	β^o^ thalassaemia trait
30. HBB:c.114G > A	Codon 37 G > A	1	1	0	H4	β^o^ thalassaemia trait
31. HBB:c.118C > T^b^	Codon 39 C > T	16	13	3	H4	β^o^ thalassaemia trait
32. HBB:c.126_129delCTTT^b^	Codon 41/42 -CTTT	2	2	0	H4 + H5	β^o^ thalassaemia trait
33. HBB:c.135delC^b^	Codon 44 -C	14	10	4	H4 + H5	β^o^ thalassaemia trait
34. HBB:c.140G > A	Hb K-Ibadan	1	1	0	H4 + H5	Hb K-Ibadan
35. HBB:c.157G > C	Hb Summer Hill	1	1	0	H5	Hb Summer Hill
36. HBB:c.251delG	Codon 82/83 -G	6	6	0	H6	β^o^ thalassaemia trait
37. HBB:c.315 + 1G > A^b^	IVS2-1 G > A	14	10	4	H6	β^o^ thalassaemia trait
38. HBB:c.316-106G > C^b^	IVS2-745 G > C	1	1	0	H8	β^+^ thalassaemia trait
39. HBB:c.316-3C > A	IVS2-848 C > A	1	1	0	H8 + H9	β^+^ thalassaemia trait
40. HBB:c.321_322insG	Codon 105/106 + G	1	1	0	H8 + H9	β^o^ thalassaemia trait
41. HBB:c.364G > C	Hb D Los Angeles	14	14	0	H9	Hb D trait
42. HBB:c.316-149_*342delinsAAGTAGA	619 bp deletion (hetero)	1	1	0	G2	β^o^ thalassaemia trait
43. HBB:c.316-149_*342delinsAAGTAGA	619 bp deletion (homo)	1	1	0	G2	β thalassaemia major
44. NG_000007.3:g.66258_184734del118477	Filipino deletion	3	2	1	G1 + G2	β^o^ thalassaemia trait
False positive by HRM		-	1	0	1	Not applicable
No mutation detected		49	44	4	-	Not applicable

Samples were analysed by HRM-PCR either after (Sample set 1; *n* = 342) or prior to (Sample set 2; *n* = 68) sequencing of the *HBB* gene. HRM-PCR correctly identified all samples with pathological sequence variations (*n* = 355). Furthermore, it was possible to correctly assign a genotype based on the HRM-PCR data (Sample set 2) for all but 10 samples (indicated by a^a^). GRACE-PCR correctly identified two known cases with 619 bp deletions and also identified a further three cases with deletions of both the promoter and third exon. Gap-PCR identified the 116 kb deletion for these three cases. The commercial β-Globin StripAssay used in our laboratory for the detection of mutations of the *HBB* gene would be able to resolve 23 (indicated by a^b^) of the 44 abnormal genotypes detected

The *CLCN7* gene was selected as a reference gene for GRACE-PCR [32]. As previously described, the *CLCN7* gene is well suited as a reference gene since it has no pseudo genes and defects in the gene result in an obvious phenotype [34, 38]. The G3 primer set was designed to target the *CLCN7* gene and generate a product with a melting temperature approximately 8 °C lower that the products of the G1 and G2 primer sets. This resulted in GRACE-PCR melting curves with two distinct steps corresponding to the melting of the reference gene product and the *HBB* product (Fig. 4). The drop in fluorescence as each product melts is proportional to the amount of the product present. Limiting the number of cycles to 26 ensured that the amount of products remained proportional to the gene copy numbers. Thus, normalized melt curve data allowed the number of gene copies to be determined by simple visual analysis (Fig. 4b).

### Initial assessment of the assay

The assay was initially assessed using 342 samples for which the *HBB* gene had been previously sequenced and included two samples positive for the 619 bp deletion. For 26 of these samples the assay did not yield the expected result, with 23 due to interference from the common synonymous SNP HBB:c.9C > T, two due to deletions and one was an abnormal melt curve obtained with primer set H1.

The initial version of the assay was not able to distinguish heterozygous Hb C (Table 3; Genotype 7, HBB:c.19G > A; *n* = 5) from the heterozygous form of the common synonymous SNP HBB:c.9C > T (Fig. 3a and b). Likewise, homozygous Hb S (Table 3; Genotype 10, HBB:c.20A > T; *n* = 18) could not be distinguished reliably from the homozygous form of the HBB:c.9C > T SNP. This issue was resolved by the use of the unlabelled probe [37, 39] designed to cover the HBB:c.19G > A and HBB:c.20A > T mutations, but not the nearby HBB:c.9C > T SNP (Fig. 1 and Table 1). This approach resulted in clear separation of the pathological mutations in the probe melt region (Fig. 3c). Repeating the HRM-PCR analysis in the presence of the unlabelled probe for the 23 samples in which a mutation was initially missed, yielded results consistent with sequencing (Table 3; columns Sequencing *versus* Sample set 1).

GRACE-PCR correctly detected the two samples known to be positive for the 619 bp deletion as having deletions of the third exon of the *HBB* gene (Table 3, genotypes 42 and 43). GRACE-PCR also indicated that two additional samples had large deletions of the *HBB* gene involving both the promoter and the third exon. Both of these unexpected cases were subsequently confirmed by Gap-PCR to be positive for the Filipino β-thalassaemia deletion (Table 3, genotype 44).

After modifying the assay to include the probe and confirming the presence of the deletions, only one of the 342 samples in the initial assessment failed to give the expected result. This sample produced an abnormal melting curve with the H1 primer set the first time it was tested. On retesting the sample was found to be wild type, this sample is listed as a false positive in Table 3.

### Validation of the assay

An additional 68 samples without prior sequence information were analysed by the assay. Sixty three samples had HRM melting curves that indicated the presence of sequence variations, other than the common HBB:c.9C > T SNP, and for one sample GRACE-PCR indicated a gene deletion. For the remaining four samples there was no evidence of a sequence change. It was noted that the HRM-PCR difference plots were highly reproducible between runs and between samples (Fig. 2; panels a *versus* b). This raised the possibility that HRM-PCR could be used not only to detect sequence variations but also reveal the actual nucleotide change. For 53 of the 63 samples in which a sequence variation was detected, it was possible to assign a provisional genotype based only on the HRM-PCR data. Subsequent sequencing of the *HBB* gene confirmed that the four samples without variation by HRM-PCR had not contained any sequence changes, and that the 63 samples identified with variations all carried pathological mutations. The sample, which tested positive by GRACE-PCR, was confirmed by Gap-PCR to be another example of the Filipino β-thalassaemia deletion. Furthermore, all 53 of the provisional genotypes assigned by analysis of the HRM-PCR data were proved to be correct. The 10 samples, for which no provisional genotype could be assigned, were found to have genotypes that were either absent, or only present in a very few cases in the initial 342 samples (Table 3; columns Sample set 2 *versus* Sample set 1).

A combination of sequencing, Gap-PCR and the commercial β-globin StripAssay showed that 361 of the 410 samples included in the study contained one or more mutations in the *HBB* gene. The assay was able to detect all 361 abnormal samples and generated just one false positive result (Table 3; columns Sequencing *versus* Sample sets 1&2). This equates to a sensitivity of 100.0 % (95 % confidence interval 98.7 - 100.0 %) and a specificity of 98.0 % (95 % confidence interval 87.8 – 99.9 %). In total, the 361 positive samples in the study accounted for 44 distinct pathological genotypes (Table 3).

## Discussion

Quantification of Hb A_2_ is routinely used for the detection of β-thalassaemia carriers. The normal range for Hb A_2_ is 2.0–3.3 % with levels above 3.5 % being considered indicative of β-thalassaemia trait [11]. The current study included 22 samples with Hb A_2_ levels greater than 3.5 %, which did not have detectable mutations or deletions of the *HBB* gene. This is in line with previous observations that false positive results can be a significant problem when screening for β-thalassaemia carriers based on Hb A_2_ levels [40]. Indeed, elevated Hb A_2_ in the absence of β-thalassaemia has recently been linked to mutations in the *KLF1* gene [14], suggesting that molecular confirmation may be required. In our laboratory, *HBB* gene mutations are routinely detected with a commercial reverse hybridization assay (ViennaLab Diagnostics). This commercial kit was designed to detect the 22 most prevalent mutations found in Indian and Middle Eastern populations. The current study identified 63 samples (15.4 %) with mutations not targeted by this kit (Table 3). This further illustrates the limitations of conventional PCR techniques when screening for β-thalassaemia trait in highly heterogeneous populations, and highlights the advantages of using a more universal assay, such as the one described here.

The first application of HRM-PCR to the *HBB* gene was described by Wittwer et al*.* [41]. This was a small-scale study designed to detect the mutations responsible for the abnormal haemoglobins Hb S and Hb C [41]. Despite the fact that they amplified approximately the same section of the gene covered by the H2 primer set in the current study, the authors did not report any problems with the HBB:c.9C > T SNP. This is presumably due to the relatively small number of samples tested (*n* = 12). In contrast, in the current study the HBB:c.9C > T SNP was encountered frequently and it was not possible to resolve all genotypes without the addition of the unlabelled probe.

Three previous studies have described HRM-PCR assays to scan the *HBB* gene in different populations. Shih et al*.* [30], Saetung et al*.* [31] and Lin et al*.* [29] used the technique to detect the most prevalent mutations in Taiwanese, Thai and Chinese populations respectively [29–31]. These assays scanned selected areas of the *HBB* gene and the assay described by Saetung et al. also included a tube for the detection of the 3.4 kb deletion. Both Shih et al*.* [30] and Lin et al*.* [29] reported problems with the HBB:c.9C > T SNP, which were resolved by placing the primer over the SNP. A possible concern with this approach is that it might result in allele dropout, particularly if a SNP is located close to the 3’ end of a primer. The primers described by Shih et al*.* [30] put the SNP close to the 3’ end, while the primer used by Lin et al*.* [29] place the SNP in the middle of the primer where it is less likely to have a significant effect. However, the HBB:c.19G > A and HBB:c.20A > T mutations are located at the 3’ end of the primer used by Lin et al*.* [29], which would probably affect the amplification of these common pathologically significant alleles. Since these mutations are rare in China [42] and were not included in the specific mutations being targeted this would not have been a significant concern when screening that population. However, these mutations are very common throughout Africa and the Middle East [43], thus the assay described by Lin et al. [29] is not well suited for screening in these regions. In contrast to overlaying the HBB:c.9C > T SNP with a primer, the approach described here of using the unlabelled probe, allowed detection of all the mutations we encountered in this area of the *HBB* gene.

Saetung et al*.* [31] avoided complications from the HBB:c9.C > T SNP by simply not scanning this region of the *HBB* gene. This approach limits the usefulness of their assay in populations where mutations in this area of the gene are common.

Other high frequency SNPs also complicated HRM analysis of the *HBB* gene. These were HBB:c.315 + 16G > C (rs10768683) and HBB:c.316-185C > T (rs1609812). We addressed this issue by substituting the respective SNP with inosine in each primer designed to cover these bases. This approach, as previously demonstrated for the *BRCA* genes [37], eliminated the detection of this high frequency SNP and provided clean HRM profiles (Fig. 2; 1a and 1b).

Until recently, deletions of the *HBB* gene were considered to be rare with only the 619 base deletion being common, accounting for around 20 % of β-thalassaemia alleles in some parts of India and Pakistan [2]. However, it has now become apparent that *HBB* gene deletions are more frequent than was previously believed. The 3.4 kb deletion has a prevalence of up to 4 % in some parts of Thailand [44] and the Filipino β-thalassaemia deletion has been described as being a common cause of β-thalassaemia in Filipinos [36, 45]. Indeed, it has been suggested that deletions involving the β-globin gene cluster account for as many as 10 % of all β-thalassaemia mutations [4].

Conventional HRM-PCR works well for the detection of point mutations and small indels, but is not a useful technique for the detection of larger gene rearrangements. Melting curve assays have been described to detect the 3.4 kb deletion of the *HBB* gene [31, 44]. However, for other deletions Gap-PCR is still commonly used. Although Gap-PCR is a simple and robust technique, it is not ideal for high volume screening, since the use of agarose gel electrophoresis for detection makes it a time consuming open tube technique. An important limitation of both Gap-PCR and the existing melt curve assays is that they can only be used to detect specific deletions with well-defined breakpoints and consequently are not suitable for the detection of novel or rare deletions. In contrast, the GRACE-PCRs included in the current study determine the copy number ratio between the *HBB* gene and the reference gene. Thus GRACE-PCR can be used to detect large rearrangements of the *HBB* gene without prior knowledge of the breakpoints. In order to maximise the number of deletions that could be detected our assay used two GRACE-PCR reactions, one targeting the promoter and the other targeting the third exon.

Allele dropout due to a SNP within a priming site is a potential risk with any PCR based assay. The use of the probe and primers containing inosine in the current assay addressed this concern for the most common high frequency SNPs associated with the *HBB* gene. However, with 868 known pathological mutations of the *HBB* gene, as well as a number of non-pathological ones, it is inevitable that there are SNPs associated with most priming sites. The risk of misinterpretation due to allele dropout is significantly mitigated by the overlapping nature of the HRM amplicons and by the use of two GRACE-PCR reactions. Furthermore, the fact that no problems due to allele dropout were encountered in the 410 samples evaluated in the study indicated that the risk is indeed small. Nevertheless, it is recommended that the possibility of allele dropout be considered when results of the assay do not correlate with other laboratory findings or the clinical picture.

The general purpose of gene scanning is to identify amplicons carrying variant sequences. The HRM-PCR profiles of the amplicons not only indicated the presence of a variation in the sequence, but also often allowed the identification of the exact nucleotide change. In the 68 blindly analysed samples all variant sequences were correctly detected. Furthermore, in 84 % of these cases the melting curves were sufficiently distinctive for the correct genotype to be assigned prior to sequencing (Table 3; columns Sample set 2 *versus* Sample set 1). We believe, as more experience is gained with this assay, provisional direct genotyping of almost all samples should be possible. The observed genotyping ability of HRM-PCR for the *HBB* gene contrasts favourably with denaturing HPLC (DHPLC) assays, as DHPLC may require either a two-step assay [46], or may require to be repeated after spiking with control DNA from a known variant [47].

When a mutation occurred in overlapping amplicon regions (Fig. 1 and Table 1) it was detected in both amplicons (Table 3). This provided additional information assisting in the assignment of the correct genotype. For example, a sample found to be positive with both primer sets H4 and H5 was likely to be either HBB:c.126_129delCTTT, HBB:c.135delC or HBB:c.140G > A, since they were the only mutations identified in the current study that occurred in this region of overlap.

Interestingly, SNPs in linkage disequilibrium with pathological mutations also provide additional information which assists in assigning the correct genotype. For example, an association between Hb Monroe (HBB:c.92G > C) and the SNP (HBB:c.-92C > G) in the *HBB* gene promoter has been reported [48]. In the current study three cases of Hb Monroe in association with HBB:c.-92C > G were found (Table 3; Genotype 22). The Hb Monroe mutation and the SNP resulted in distinctive melting curves in amplicons obtained with primer sets H3 and H1, respectively.

No mutations were found in the region of the *HBB* gene scanned by primer sets H7, H10 and H11, indicating that mutations in this part of the gene are comparatively rare in the population studied (Table 3). It is interesting to note that even in the highly heterogeneous population of the UAE, 91 % of positive cases could be detected using just 5 of the 13 primer sets. This suggests that a stepwise approach could be used starting with high mutation frequency amplicons, thus reducing the number of PCR reactions required to obtain results.

The combination of the HRM-PCR and GRACE-PCR assays theoretically cover 97 % of the mutations listed in the HbVar database [3]. This compares favourably with previously described assays that range from 35 to 90 % theoretical coverage [29, 30]. An example of a mutation that would not be detected by the current assays is HBB:c.203_204delTG [35] due to lack of overlap between primer sets H5 and H6. Similarly, HBB:c.316-146 T > G and HBB:c.316-125A > G [3] would not be detected due to lack of overlap between primer sets H7 and H8.

Only negative (wild type) controls were included in the current study, since all samples screened were also sequenced. However, when using the assays for clinical testing, the inclusion of appropriate positive and negative controls with each run is highly recommended. Such controls could be genomic DNA from confirmed cases or plasmids [29].

In our laboratory, the costs for scanning the *HBB* gene with the combined HRM-PCR/GRACE-PCR assay were approximately one eighth the cost of sequencing. Given that in at least 84 % of cases HRM-PCR allowed the assignment of a provisional genotype, this represents an opportunity for significant cost savings. Should sequencing be required to evaluate an unexpected melt curve, this can be performed by directly sequencing the positive HRM amplicon [20], which is still cheaper and faster than sequencing the entire *HBB* gene. In addition, the HRM-PCR / GRACE-PCR approach allows the detection of large deletions which are not readily detected by sequencing. In the current study, the samples were manually pipetted and processed in batches of 72 PCR reactions. Since both HRM-PCR and GRACE-PCR lend themselves to automation, robotic reaction setup is possible, and if a micro-titre plate based PCR system was used, up to 384 reactions could be run simultaneously on a single plate. Although we used different annealing temperatures, all the primers used for HRM-PCR will perform adequately at 55 °C, which would simplify batch processing if micro-titre plates were adopted.

In conclusion, the current study has resulted in the development of a robust assay for the detection of pathological *HBB* gene mutations. The approaches used for handling the high frequency SNPs mean that the assay is universally applicable, rather than being population specific. The inclusion of GRACE-PCR targeting both the gene promoter and third exon means that the assay has the ability to detect a wide range of large deletions of the *HBB* gene. The assay has sensitivity and specificity comparable to sequencing and was able to detect all mutations in the samples tested. Furthermore, this assay can be run at a fraction of the cost of a full sequencing approach as 84 % of positive samples could be provisionally genotyped. The assay has provided useful data on the spectrum of *HBB* gene mutations in the highly heterogeneous population of the United Arab Emirates. In future it should be able to serve as a screening test for suspected β-thalassaemia carriers and variant haemoglobins.

## Abbreviations

ARMS: Amplification Refractory Mutation System
ASO: Allele specific oligonucleotide
CE: Capillary electrophoresis
*CLCN7*: Chloride channel voltage sensitive 7 gene
DGGE: Denaturing gradient gel electrophoresis
DHPLC: Denaturing high performance liquid chromatography
EDTA: Ethylenediaminetetraaceitc acid
GRACE: Gene ratio assay copy enumeration
*HBB*: Haemoglobin beta gene
*HBD*: Haemoglobin delta gene
HPLC: High performance liquid chromatography
HRM: High resolution melting
*KLF1*: Krüppel-like Factor 1 gene
PCR: Polymerase chain reaction
SNP: Single nucleotide polymorphism
SSCP: Single Strand Conformational Polymorphism
WHO: World Health Organization
